# Editorial: Technological Advances for Gait and Balance Assessment

**DOI:** 10.3390/bioengineering13060651

**Published:** 2026-05-31

**Authors:** Alessandro Zampogna, Florenc Demrozi, Silvia Del Din

**Affiliations:** 1 Department of Human Neurosciences, Sapienza University of Rome, 00185 Rome, Italy; 2 IRCCS Neuromed Institute, 86077 Pozzilli, Italy; 3 Department of Electrical Engineering and Computer Science, University of Stavanger, 4021 Stavanger, Norway; florenc.demrozi@uis.no; 4 Translational and Clinical Research Institute, Newcastle University, Newcastle upon Tyne NE4 5PL, UK; silvia.del-din@newcastle.ac.uk; 5 Newcastle Biomedical Research Centre (BRC), National Institute for Health and Care Research (NIHR), Newcastle University, Newcastle upon Tyne NE4 5PL, UK

Gait and balance disorders are a major source of disability worldwide, affecting a broad range of neurological and systemic conditions, such as Parkinson’s disease, stroke, multiple sclerosis, and musculoskeletal disorders. Across these conditions, disruption of the complex neural and biomechanical systems underlying locomotion and postural control leads to heterogeneous clinical manifestations, which nonetheless converge on common and clinically meaningful outcomes, including increased risk of falls, progressive loss of independence, and reduced quality of life [1]. The societal and economic burden associated with these disorders is substantial, representing a significant public health concern. Indeed, fall-related injuries, which are one of the most relevant clinical consequences of gait and balance impairment, account for approximately USD 80,000,000,000 in annual healthcare costs in the United States, reflecting not only direct healthcare costs but also long-term care needs and loss of productivity [2,3]. Despite their clinical relevance, traditional assessment methods like clinical scales and questionnaires remain constrained by intrinsic limitations, including subjectivity, low sensitivity to subtle changes, and limited ecological validity. While widely used, standard clinical assessments are sporadic and provide only a snapshot of the current function, failing to capture the dynamic and context-dependent nature of gait and balance performance in daily life [4]. In this context, the rapid evolution of biomedical technologies offers a concrete opportunity to redefine how these functions are assessed and managed [5,6]. This Special Issue provides a focused overview of recent advances across clinical neurology, biomedical engineering, and data science, underscoring how objective, quantitative, and scalable tools are progressively being integrated into both research and clinical practice.

A central theme emerging from several contributions of the Special Issue is the growing role of digital health technology. Wearable systems, typically based on inertial measurement units, enable continuous, unobtrusive monitoring of movement in real-world settings, capturing spatiotemporal gait parameters and other motor features with high temporal resolution. By using wearables, Boschi et al. [7] showed that sensor-derived gait metrics, when combined with clinical variables, improve the identification of patients with Parkinson’s disease (PD) at risk of short-term gait deterioration, achieving superior predictive performance compared with clinical data alone. This finding supports the integration of digital outcomes into clinical decision-making frameworks, with potential implications for personalized management strategies in PD. Extending beyond neurological conditions, Sarvestan et al. [8] also demonstrated that accelerometer-based monitoring is feasible, well accepted, and robust for assessing mobility, physical activity, and sleep in patients with rheumatoid arthritis, even under conditions of reduced data sampling. Collectively, these studies highlight the potential of wearable technologies to provide continuous and ecologically valid measurements, as data are acquired directly within the patient’s habitual environment during daily activities.

Another key area emphasized in this Special Issue is the integration of artificial intelligence and advanced computational approaches, aimed at automating the analysis of large and complex datasets that would otherwise be difficult to manage in routine clinical settings. In this regard, Lu et al. [9] developed an automated framework for estimating Berg Balance Scale scores in patients affected by PD or stroke using wearable device data and an attention-based deep learning model, achieving high accuracy and offering a scalable alternative to traditional, operator-dependent assessments. Notably, the estimation of balance performance was based on gait-related signals, thus further underscoring the close interplay between locomotion and postural control and opening the possibility for remote evaluation of functions (i.e., balance) that typically require in-person clinical interaction. Similarly, Rezapour et al. [10] combined linear mixed-effects models and machine learning techniques to characterize recovery trajectories following lower extremity fractures, demonstrating how quantitative gait parameters can inform individualized rehabilitation strategies and improve outcome prediction. Overall, these contributions support a transition toward data-driven, personalized models of care, extending beyond hospital-based settings into telemedicine and remote monitoring contexts. In parallel, advances in motion analysis technologies are improving the accessibility and scalability of gait assessment. Molteni and Andreoni [11] evaluated a markerless motion capture approach based on computer vision algorithms in children with cerebral palsy, demonstrating good agreement with gold-standard optoelectronic systems for both spatiotemporal parameters and kinematic measures, particularly in the sagittal plane. These findings suggest that low-cost, markerless solutions may represent a viable alternative for both clinical and remote applications, reducing reliance on specialized laboratory infrastructures and facilitating broader implementation, including in resource-limited settings.

Importantly, this Special Issue also highlights the relevance of patient-centered perspectives in the adoption of emerging technologies. Geßner et al. [12] explored patient-reported experiences with sensor-based motor assessments in individuals with multiple sclerosis, reporting high overall acceptability and perceived utility, while also identifying key factors influencing usability and engagement. This underscores that successful implementation of technological innovations in clinical practice depends not only on technical performance, but also on alignment with patient expectations, preferences, and real-world usability. Taken together, the contributions included in this Special Issue reflect a clear shift toward more objective, continuous, and individualized assessment of gait and balance. At the same time, several challenges remain. Many studies are based on relatively small and heterogeneous cohorts, and further validation across diverse populations and clinical settings is required. In addition, issues related to methodological standardization, model interpretability, and integration into existing clinical workflows will need to be addressed to enable widespread adoption. Indeed, despite rapid technological advances, their translation into routine clinical practice remains currently limited.

In conclusion, this Special Issue highlights the transformative potential of emerging technologies in the assessment of gait and balance both in neurological conditions and musculoskeletal disorders. By combining wearable sensors, advanced analytical methods, and patient-centered approaches, these innovations are paving the way toward more precise, scalable, and personalized management of movement disorders. Future research should prioritize large-scale validation, longitudinal monitoring, and the development of integrated digital health ecosystems to facilitate the translation of these advances from laboratory and experimental settings into routine clinical practice.

## Data Availability

Data sharing is not applicable.
